# Genome-wide identification and functional analysis of the TIFY gene family in the response to multiple stresses in *Brassica napus* L.

**DOI:** 10.1186/s12864-020-07128-2

**Published:** 2020-10-22

**Authors:** Xin He, Yu Kang, Wenqian Li, Wei Liu, Pan Xie, Li Liao, Luyao Huang, Min Yao, Lunwen Qian, Zhongsong Liu, Chunyun Guan, Mei Guan, Wei Hua

**Affiliations:** 1 Southern Regional Collaborative Innovation Center for Grain and Oil Crops in China, Hunan Agricultural University, Changsha, 410128 Hunan China; 2 Oil Crops Research, Hunan Agricultural University, Changsha, 410128 Hunan China; 3 Hunan Branch of National Oilseed Crops Improvement Center, Changsha, 410128 Hunan China; 4 Oil Crops Research Institute of the Chinese Academy of Agricultural Sciences, Key Laboratory of Biology and Genetic Improvement of Oil Crops, Ministry of Agriculture and Rural Affairs, Wuhan, 430062 China

**Keywords:** *Brassica napus*, TIFY, Abiotic stresses, Freezing, *Sclerotinia sclerotiorum*, Hormone, MeJA

## Abstract

**Background:**

TIFY is a plant-specific protein family with a diversity of functions in plant development and responses to stress and hormones, which contains JASMONATE ZIM-domain (JAZ), TIFY, PPD and ZML subfamilies. Despite extensive studies of TIFY family in many other species, TIFY has not yet been characterized in *Brassica napus*.

**Results:**

In this study, we identified 77, 36 and 39 TIFY family genes in the genome of *B. napus*, *B. rapa* and *B. oleracea*, respectively. Results of the phylogenetic analysis indicated the 170 TIFY proteins from *Arabidopsis*, *B. napus*, *B. rapa* and *B. oleracea* could be divided into 11 groups: seven JAZ groups, one PPD group, one TIFY group, and two ZIM/ZML groups. The molecular evolutionary analysis showed that TIFY genes were conserved in Brassicaceae species. Gene expression profiling and qRT-PCR revealed that different groups of *BnaTIFY* members have distinct spatiotemporal expression patterns in normal conditions or following treatment with different abiotic/biotic stresses and hormones. The *BnaJAZ* subfamily genes were predominantly expressed in roots and up-regulated by NaCl, PEG, freezing, methyl jasmonate (MeJA), salicylic acid (SA) and *Sclerotinia sclerotiorum* in leaves, suggesting that they have a vital role in hormone signaling to regulate multiple stress tolerance in *B. napus*.

**Conclusions:**

The extensive annotation and expression analysis of the *BnaTIFY* genes contributes to our understanding of the functions of these genes in multiple stress responses and phytohormone crosstalk in *B. napus*.

## Background

Plants often encounter various abiotic and biotic stresses during their life cycles, such as salinity, dehydration, extreme temperature and infection by pathogens. Plant hormones, such as abscisic acid (ABA), jasmonic acid (JA), ethylene (ET) and salicylic acid (SA) act as key defense signals in the regulation of various abiotic and biotic stress responses [1–4]. There are many types of stress-related transcriptional factors and transcriptional regulators that function as important components in the crosstalk between hormone signals to regulate tolerance to stress in plants [5, 6].

TIFY is a plant-specific protein family with a diversity of functions in plant development and responses to stress and hormones, which is characterized by a highly conserved TIFY motif (TIF[Y/F]XG) that contains the JAZ (JASMONATE ZIM-domain), TIFY, PPD (PEAPOD) and ZIM/ZML (Zinc-finger Inflorescence Meristem: ZIM and ZIM-like) subfamilies [7, 8]. The JAZ subfamily contains a TIFY domain and a JA-associated (Jas, CCT-2) domain. The ZIM/ZML subfamily contains a TIFY domain, a CCT (CONSTANS, CO-like and TOC1) domain and a GATA zinc-finger domain [7, 9–11]. The Jas and CCT domains have a similar N-terminus. The TIFY subfamily proteins have only a TIFY domain. PPD subfamily proteins contain a PPD domain, a TIFY domain and a partial Jas domain that lack PY (Proline-Tyrosine) at their C-terminus region (Chung et al., [10, 12]).

In *Arabidopsis*, ZML1 and ZML2 are involved in the cry1-mediated photoprotective response*,* and the overexpression of *AtTIFY1* (*ZIM*) produced elongated petioles and hypocotyls [13, 14]. AtTIFY4a (PPD1) and AtTIFY4b (PPD2) are involved in the synchronization of leaf growth [15, 16]. The JAZ protein is a key repressor in the plant-specific JA signaling pathway [6, 9, 11, 17, 18], which plays an important role in the resistance of plants to necrotrophic and hemibiotrophic fungal pathogens, such as *Botrytis cinerea* [19], *Fusarium oxysporum* [20], *Sclerotinia sclerotiorum* [21] and *Verticillium dahlia* [22]. JAZ proteins that contain a Jas domain can be recognized by SCF^COI1^ and degraded by the 26S proteasome [18]. The constitutive expression of JAZ proteins lacking a Jas domain confers insensitivity to JA [12, 17, 18, 23]. JAZ proteins interact with various functional types of proteins, such as transcription factors, regulators, bacterial/fungal effectors, and subsequently regulate the accumulation of anthocyanin and initiation of trichomes [24, 25], cotton fiber development [26], stamen development [27], flowering time [28, 29], tolerance to pathogen infections [22, 30, 31], insect herbivory [32] and abiotic stresses [33, 34]. For example, GhJAZ2 interacts with and represses the basic helix-loop-helix (bHLH) transcriptional factors GhbHLH171, which subsequently reduces the level of resistance to *V*. *dahlia* in cotton [22]. OsJAZ1 interacts with and represses OsbHLH148, which subsequently reduces tolerance to drought in rice [33]. JAZ1/JAZ4 physically interacts with INDUCER OF CBF EXPRESSION 1 (ICE1) and represses the transcriptional function of ICE1, thereby attenuating the expression of C-REPEAT BINDING FACTORS (*CBFs*) [34]. The overexpression of *JAZ1* or *JAZ4* represses the responses of *Arabidopsis* to freezing stress [34].

*B. napus* (genome AnAnCnCn) is a crucial oil crop in China and worldwide, which was formed by recent alloploidy between ancestors of *B. rapa* (genome ArAr) and *B. oleracea* (genome CoCo) [35]. Since *B. napus* is sensitive to environmental stress during all stages of growth and development, the production and quality of *B. napus* worldwide is deleteriously restricted by abiotic stresses and infection with *S. sclerotiorum*. Despite extensive studies of the TIFY family in many other species, including *B. rapa* and *B. oleracea* [36–51], TIFY has not yet been characterized in *B. napus*.

To investigate the potential roles of *B. napus* TIFY proteins in response to phytohormone and multiple stresses, we identified 77 *TIFY* genes in *B. napus*. The putative 170 TIFY proteins from *Arabidopsis*, *B. napus*, *B. rapa* and *B. oleracea*, were classified into 11 groups: seven JAZ groups, one TIFY group, one PPD group, and two ZIM/ZML groups. In addition, *TIFY* gene structures, chromosomal locations, and conserved motifs were subsequently analyzed. Gene expression analysis revealed that different groups of *BnaTIFY* gene family members have distinct spatiotemporal expression patterns in normal conditions or following treatment with different abiotic/biotic stresses and hormones. The JAZ subfamily members were predominantly expressed in *B. napus* roots, strongly induced by NaCl and PEG in roots and induced by MeJA, SA, freezing and *S. sclerotiorum* in leaves, suggesting that they have a vital role in hormone signaling to regulate multiple stress tolerance in *B. napus*.

## Results

### Identification of TIFY in *B. napus*, *B. rapa* and *B. oleracea*

To identify all the TIFY proteins in *B. napus, B. rapa* and *B. oleracea*, we performed a BLASTp search against the annotated proteins of *B. napus, B. rapa* and *B. oleracea* in Ensembl Plants (http://plants.ensembl.org/index.html) using the 18 *Arabidopsis* AtTIFY protein (12 JAZ, 1 TIFY, 2 PPD and 3 ZML) sequences as queries. A set of 77 (52 JAZ, 6 TIFY, 4 PPD and 15 ZML), 36 (26 JAZ, 2 TIFY, 2 PPD and 6 ZML) and 39 (27 JAZ, 5 TIFY, 2 PPD and 5 ZML) TIFY proteins were identified in *B. napus, B. rapa* and *B. oleracea*, respectively (Additional file 1). There are 70, 36 and 36 proteins that contain the TIFY domain in *B. napus, B. rapa* and *B. oleracea*, respectively, through simultaneous consideration of the conservation of the TIFY domain from an InterPro and Conserved Domain (CD)-search of the NCBI database. The predicted subcellular localizations of these proteins using ProtComp Version 9.0 revealed that most of the TIFY proteins might be located in the nucleus, with the exception of 14 proteins, including all 12 members that are homologous to TIFY9 and two members that are homologous to TIFY8 (Additional file 1). The TIFY genes in *B. napus, B. rapa* and *B. oleracea* were named based on the homology of their coding proteins to *Arabidopsis* TIFY proteins (Additional file 1).

Among the 105 JAZ subfamily proteins in *B. napus, B. rapa* and *B. oleracea*, 99 were typical JAZ proteins that contained both the TIFY and Jas domains, and 41 of the JAZ have an N-terminal (NT) domain near the N-terminus in addition to the TIFY domain. All 41 NT-TIFY-Jas type JAZ proteins were homologous to AtJAZ5/6 and AtJAZ1/2. Two pairs of BnaJAZ proteins (BnaJAZ7-A3/C3 and BnaJAZ3/4-C2/A3) contained only a single Jas domain, while BnaJAZ2-A1–1 contained only an NT domain, which was located 81 bp upstream of a TIFY-Jas type BnaJAZ (BnaJAZ2-A1–2). Thus, BnaJAZ2-A1–1 and BnaJAZ2-A1–2 may be divided from a NT-TIFY-Jas type BnaJAZ. All eight BnaPPD members contained both TIFY and Jas domains. Among the 26 members of ZML subfamily, 20 were typical ZML proteins that contained (TIFY)-(CCT)-(GATA zinc-finger) domains in order, while six members contained only a single TIFY domain. Among the TIFY subfamily, eight members contained only a single TIFY domain, and five members that have no domain had been reported (Additional file 1).

### Phylogenetic analysis of the TIFY proteins

**Fig. 1:**
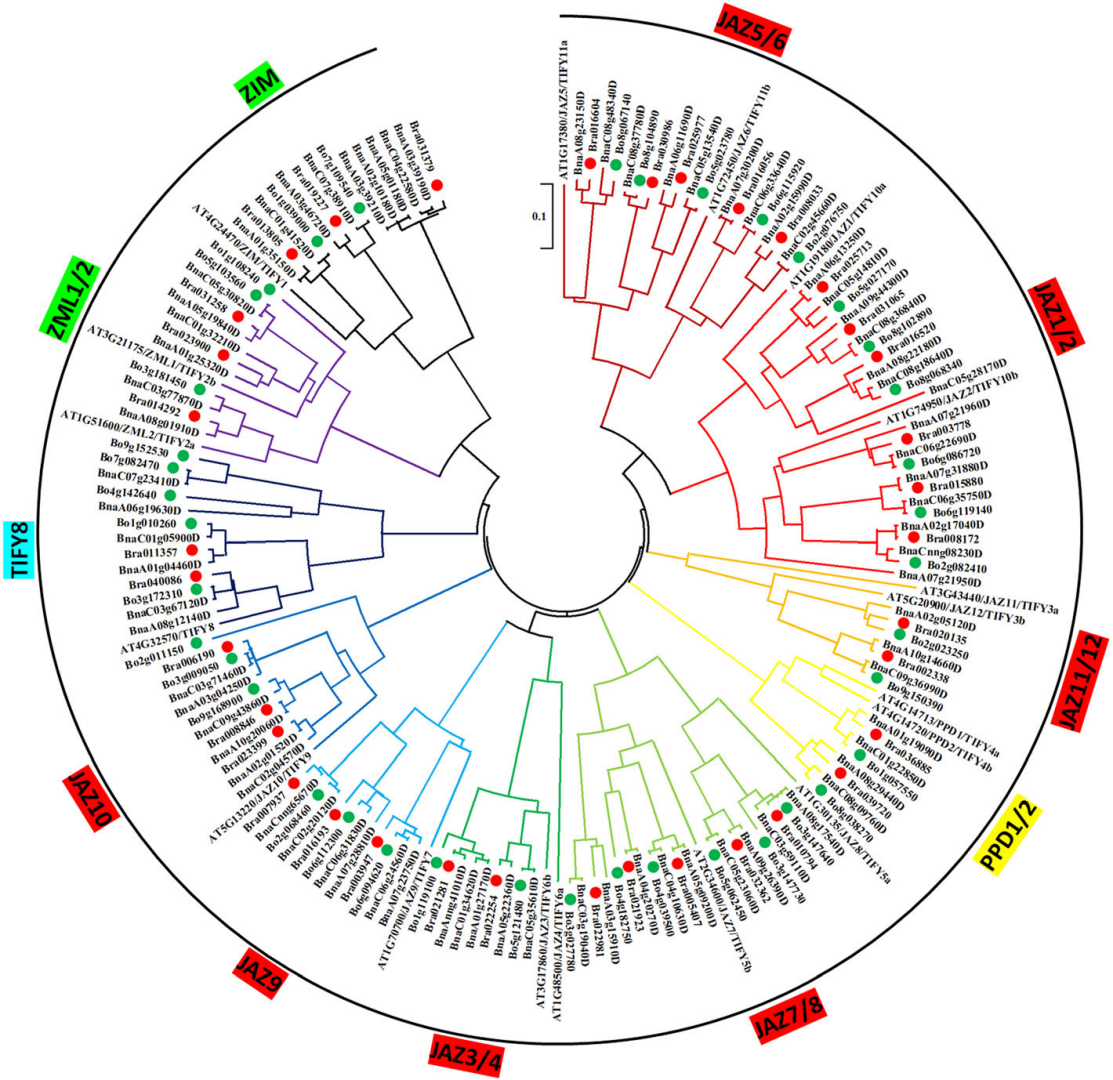
Phylogenetic analysis of 170 TIFY proteins from *B. napus* (77), *B. rapa* (36), *B. oleracea* (39) and *Arabidopsis* (18). A neighbor-joining phylogenetic tree was generated by MEGA X with full-length TIFY sequences (1000 bootstrap replicates). The resulting 11 groups classified in four resulting subfamilies (JAZ, PPD, TIFY and ZIM/ZML were highlighted by red, yellow, turquoise and green, respectively) are labeled

To explore the classification and evolutionary characteristics of the TIFY proteins, an un-rooted phylogenetic tree based on the 170 protein sequences of *B. napus* (77), *B. rapa* (36), *B. oleracea* (39) and *Arabidopsis* (18) TIFY was constructed in MEGA X (Fig. 1). According to this phylogenetic analysis, the BnaTIFY proteins could be divided into 11 groups: seven JAZ groups, one PPD group, one TIFY group, and two ZIM/ZML groups. Interestingly, although there were six members in the ZIM group that consisted of only a single TIFY domain, a phylogenetic analysis revealed that they were not divided into TIFY groups (Additional file 1 and Fig. 1).

According to the homologous gene sets among the A_r_ (*B. rapa*), C_o_ (*B. oleracea*), A_n_- and C_n_-subgenomes of *B. napus*, 37 A_r_-C_o_-A_n_-C_n_ pairs were identified among the 152 *Bna/Br/BoTIFY* genes (Additional file 1 and Fig. 1). One *B. rapa JAZ* gene *BrJAZ5–2* (*Bra030986*) lacked a homologous gene in the A_n_-subgenomes of *B. napus*, and 5 *B. oleracea TIFY* genes (*BoJAZ11/12–1, BoJAZ7–2, BoJAZ8–1-2, BoTIFY8–4, and BoTIFY8–5*) lacked a homologous gene in the C_n_-subgenomes of *B. napus*. Seven *B. napus TIFY* genes (*BnaTIFY8-A3, BnaJAZ1-C4, BnaJAZ3/4-A3, BnaZIM1-C3, BnaZIM1-A4, BnaZIM1-A5, and BnaZIM1-A6*) lacked a homologous gene in *B. rapa* or *B. oleracea* (Additional file 1 and Fig. 1).

The *B. napus* An- and Cn-subgenomes were largely collinear to the corresponding diploid Ar and Co genomes, and most of the An-Ar and Cn-Co orthologous gene pairs demonstrated similar chromosomal locations (Additional file 2) [35]. A total of 77 *BnaTIFY* genes were distributed unevenly among the 19 chromosomes of *B. napus* and were equally distributed on the A_n_- and C_n_-subgenomes (40 and 37 genes, respectively) (Additional file 3). The distribution of *TIFY* genes in *B. rapa* and *B. oleracea* were similar to the distribution of orthologous *BnaTIFY* genes in the *B. napus* An- and Cn-subgenomes, respectively (Additional file 3).

### Motif analysis (MEME) and gene structural analysis of TIFY

**Fig. 2:**
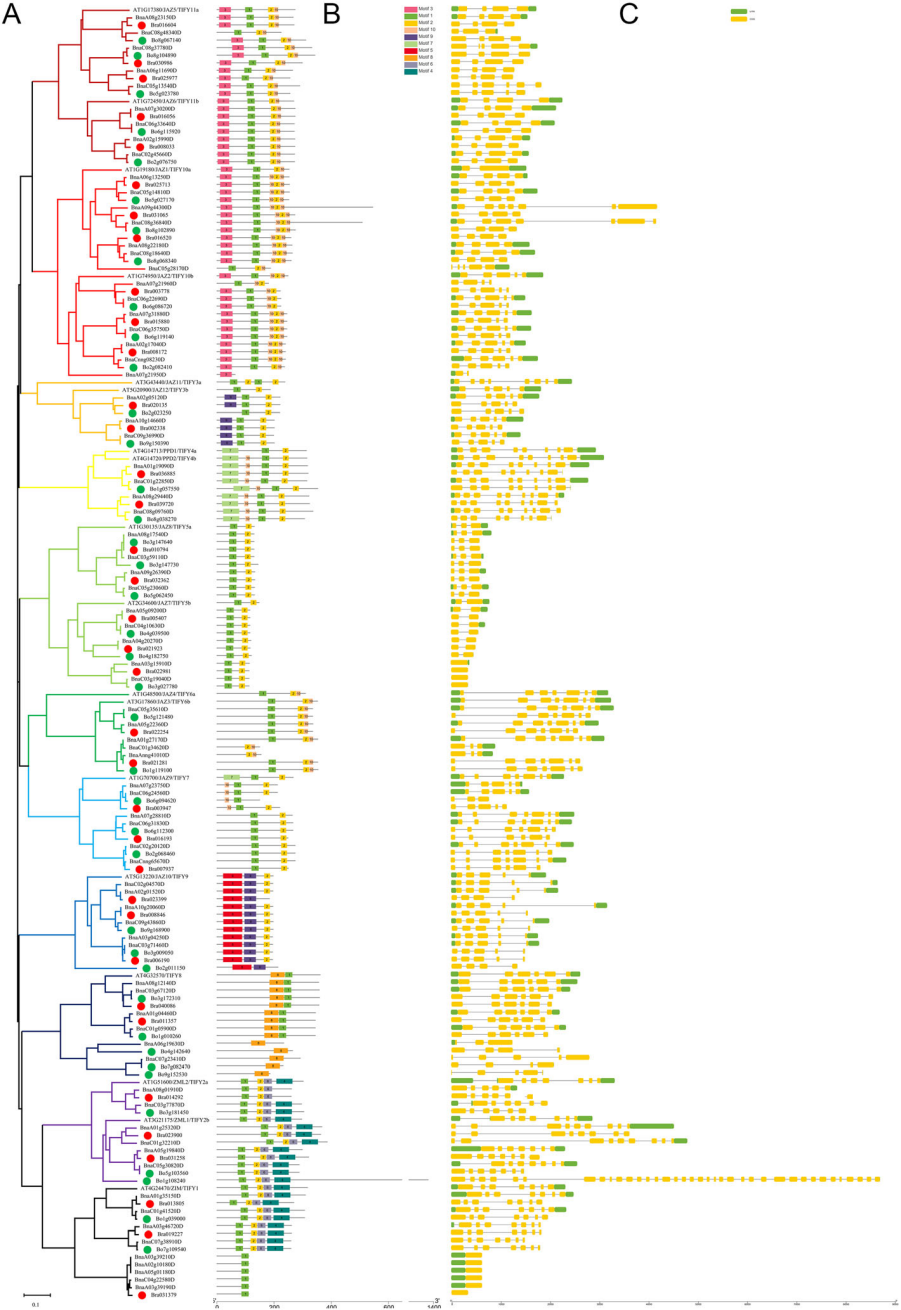
Phylogenetic analysis (**a**), MEME analysis (**b**) and gene structures (**c**) of 170 TIFY from *B. napus* (77), *B. rapa* (36), *B. oleracea* (39) and *Arabidopsis* (18)

The protein sequences were analyzed using MEME to analyze the conserved motifs in the TIFY proteins, and 10 motifs were detected in 170 TIFY proteins from *B. napus* (77), *B. rapa* (36), *B. oleracea* (39) and *Arabidopsis* (18): motif 1 (TIFY domain), motif 2 (N-terminus of the Jas/CCT domain), motif 3 (NT domain), motif 4 (GATA zinc-finger domain), motif 5 (unknown domain 1), motif 6 (C-terminus of the CCT domain), motif 7 (PPD domain), motif 8 (unknown domain 2), motif 9 (TIFY domain) and motif 10 (C-terminus of the Jas domain) (Additional file 4). Motif2-motif10 forms the Jas domain, and Motif2-motif6 forms the CCT domain. According to Additional files 1 and 4 and Fig. 2b, two isoforms (motif 1 and motif 9) of the TIFY domain were identified. The motif1-type TIFY domain was the most conserved and was located in 145 TIFY members, while the motif 9-type TIFY domain was located in all 13 members in TIFY9 group. Six members from the TIFY3 group contained both motif 1 and motif 9. Additionally, the NT domain (motif 3) was only found in the TIFY10 and TIFY11 groups; motif 5 (unknown domain 1) was only found in the TIFY9 group, while motif 8 (unknown domain 2) was only found in the TIFY8 group (Additional file 1 and Fig. 2b).

To study the structural diversity of *TIFY* genes, the exon/intron organization of individual *TIFY* genes was analyzed. The number of introns ranges from 0 to 36 in *TIFY* genes. In general, members from a group have a similar gene structure. The detailed gene structures are shown in Fig. 2c. Compared with the homologous genes (*BrZML1–1, BnaZML1-A1* and *BnaZML1-C1*), the *BoZML1–1* gene is extraordinarily long. As shown in Additional file 5, in the homologous chromosomal regions of *BoZML1–1*, all have two tandem genes (*BrZML1–1-Bra023899, BnaZML1-A1-BnaA01g25310D* and *BnaZML1-C1-BnaC01g32200D*) in *B. rapa* and the A_n_- and C_n_-subgenomes of *B. napus*, respectively. A sequence analysis showed that the *BoZML1–1* start codon region and its nearby areas were the same as that in third exon region of *BnaZML1-C1*. Additionally, in *B. rapa*, there were no SNPs in the regions homologous to the *BnaZML1-C1* start/stop codon region and their adjacent areas (Additional file 6). The only difference was that there was an insertion (ATACG) before the first codon of the seventh exon (the region homologous to the first exon of *BnaC01g32200D*) (Additional file 6). We hypothesized that it could be the primary cause of different splice in region of *BoZML1–1*.

The core sequences of three domains in 77 BnaTIFY proteins were analyzed to explore the TIFY, Jas and CCT domains in TIFY proteins. As shown in Additional file 7, Motif 9 in the TIFY9 group also has the core sequence (TIFYXG) of TIFY domain, while there was a deletion (14th AA) in Motif 9 compared with the Motif1-type TIFY domain. Additionally, unlike Motif 9 in the TIFY9 group, Motif 9 in the TIFY3 group lacks the core sequence of TIFY domain. Notably, there were variations in the core sequence of Motif 1-type TIFY domain. The core sequence of TIFY domain in TIFY11 and TIFY3 groups was TIFF[G/R][G/S], while it was T[I/L]SF[Q/R/C]G in the ZIM/ZML group.

As shown in Additional file 8, Motif 2 was the common N-terminus of Jas and CCT domains, while motif 10 contained the core two conservative amino acid residues (PY) of the C-terminal region of Jas domain. Motif 6 contained the C-terminal region of CCT domain. Although the TIFY3/5/7/9 groups were not contained motif 10, they all contained the conservative PY residues. The PPD subfamily lacked motif 2 and the conservative PY residues. The ZIM/ZML subfamily contained motif 2 and motif 6.

To obtain a more thorough understanding of the evolutionary constraints that act on the TIFY gene family in *Arabidopsis*, *B. rapa*, *B. oleracea* and *B. napus*, all of the aligned sequences of TIFY cDNA and their cDNA region of the TIFY domain were used to calculate the number of nonsynonymous substitutions per nonsynonymous site (*Ka*), the number of synonymous substitutions per synonymous site (*Ks*) and the *Ka/Ks* ratio. The *Ka/Ks* ratio was < 1 for majority of the TIFY orthologues gene pairs, whether the whole cDNA sequences or only the TIFY domain regions were examined (Additional file 9). However, the TIFY domain regions had lower average *Ka/Ks* ratios than the whole cDNA sequences (Additional file 9). These results indicate that the majority of TIFY genes experienced purifying selection, and the TIFY domains were strongly controlled in evolution.

### Expression profiling of *BnaTIFY* genes in different tissues and under abiotic/biotic stress and phytohormone treatments

**Fig. 3:**
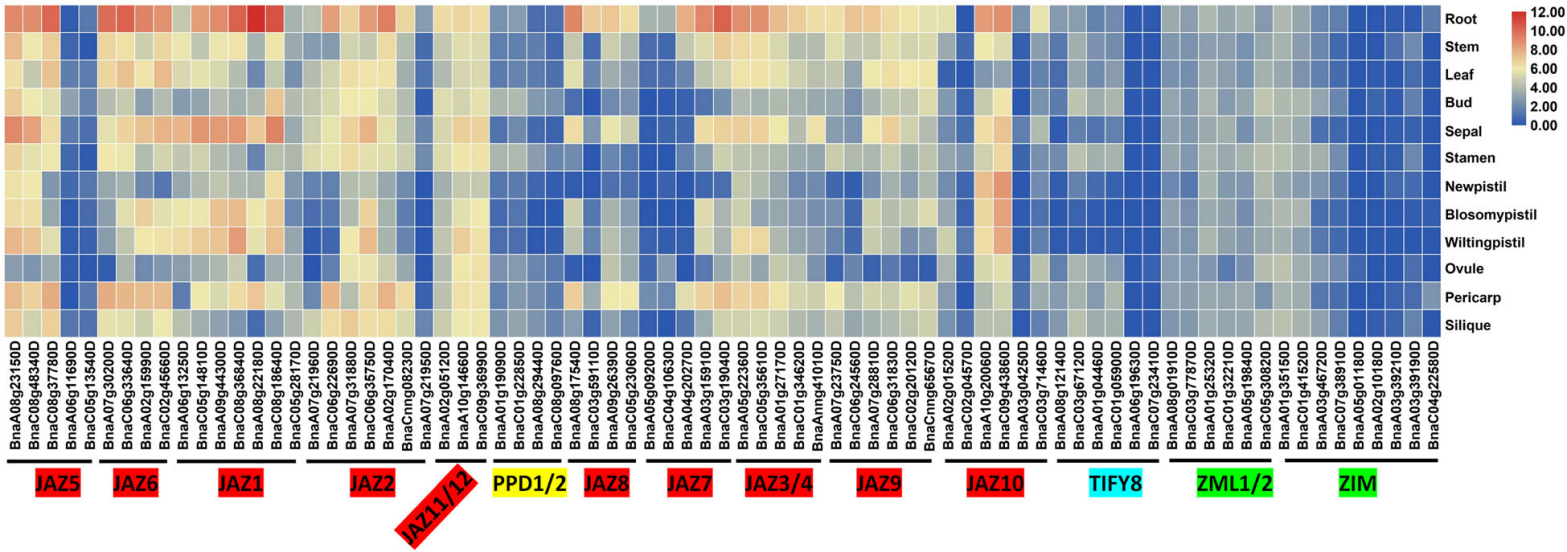
Expression of *BnaTIFY* genes in different tissues and organs. The expression levels of each gene (Log_2_ (FPKM values)) in each sample, as indicated by different color rectangles

A heatmap was drawn based on the RNA-Seq data (Additional file 10) from 12 *B. napus* tissues (roots, stems, leaves, buds, sepals, stamens, new pistils, mature pistils, wilting pistils, ovules, pericarps and siliques) to examine the patterns of expression of *BnaTIFY* in different *B. napus* tissues and organs. As shown in Fig. 3, the *BnaTIFY* genes were differentially expressed among the group in 12 tissues. All of the JAZ subfamily members were generally transcribed at higher levels than those of the other three subfamilies and were predominantly expressed in *B. napus* roots.

**Fig. 4:**
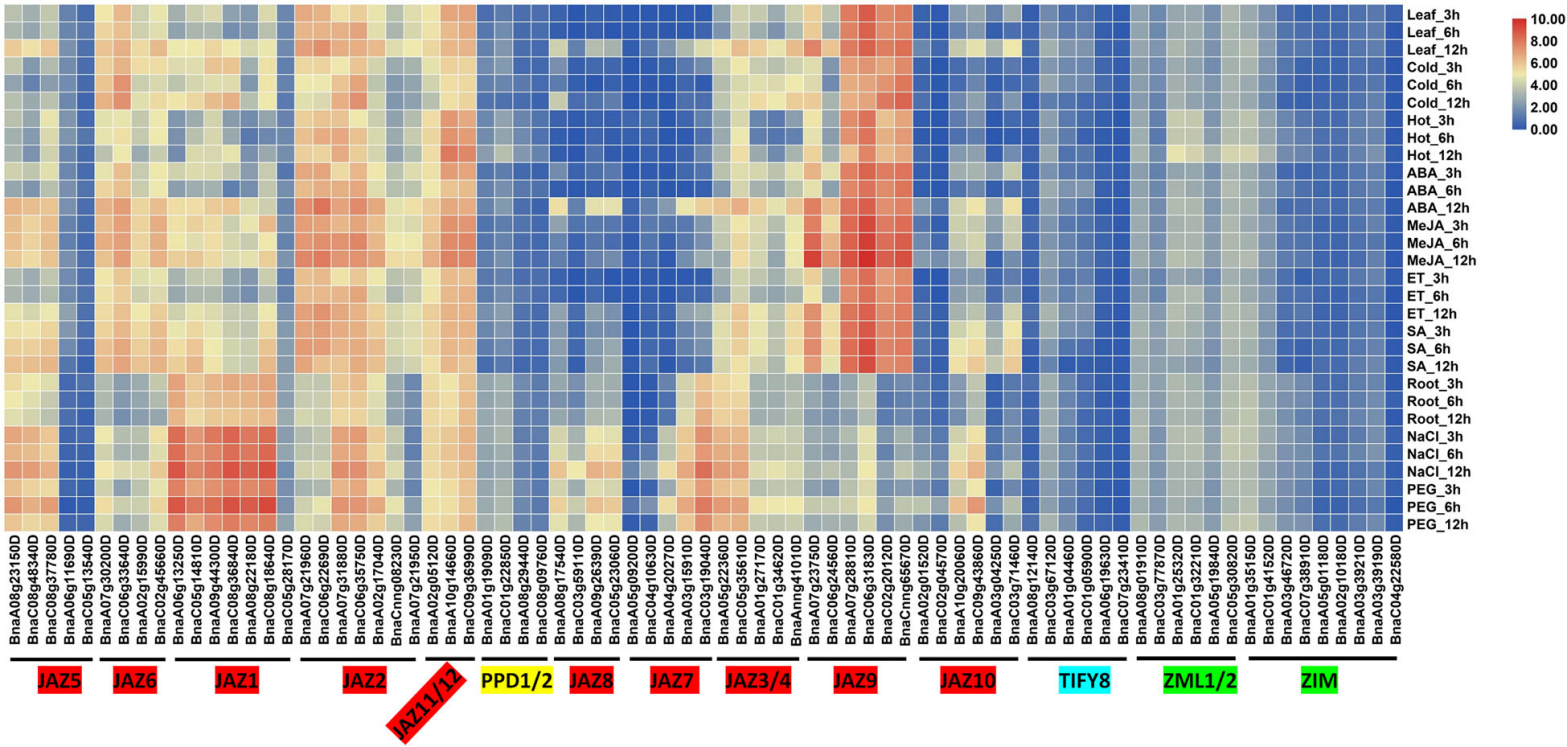
Expression of *BnaTIFY* genes under different abiotic stresses and plant hormones treatments. The expression levels of each gene (log_2_ (TPM values)) in each sample, as indicated by different color rectangles

To characterize the abiotic-responsive *BnaTIFY* genes, the transcripts per million (TPM) value of each gene (Additional file 11) in leaves treated with cold, heat, ABA, methyl jasmonate (MeJA), ethylene, and SA, and in roots treated with NaCl and PEG were determined. As shown in Fig. 4, most of the JAZ subfamily members were induced by MeJA and SA in leaves and by NaCl and PEG in roots, while the PPD and ZML subfamilies were induced by heat treatment. Interestingly, almost all of the JAZ subfamily members were regulated by the circadian clock.

**Fig. 5:**
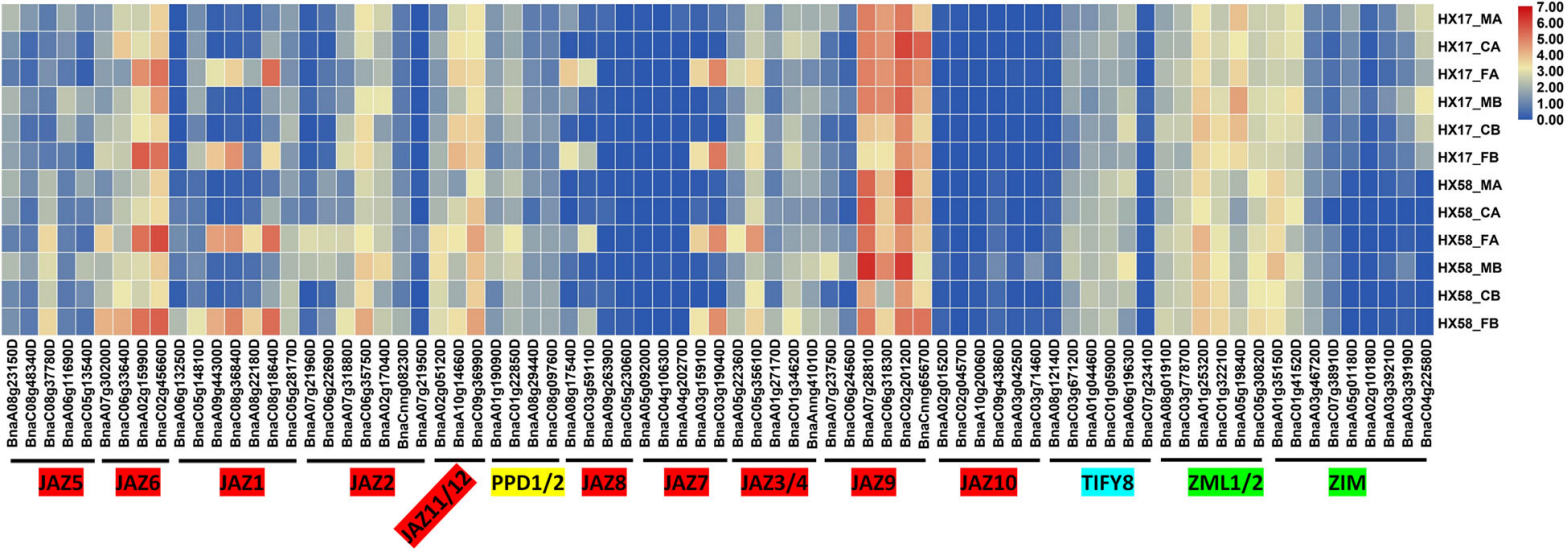
Expression of *BnaTIFY* genes under different low-temperature treatments. The expression levels of each gene (log_2_ (FPKM values)) in each sample, as indicated by different color rectangles

To detect the response of *BnaTIFY* genes to different low temperature stresses, the reads per kilobase of transcripts per million mapped reads (RPKM) values of each gene (Additional file 12) in leaves treated with chilling and freezing with or without cold acclimation were determined using the data from GEO (GSE129220) [52]. As shown in Fig. 5, the JAZ subfamily (TIFY11b, TIFY10a, TIFY3, TIFY5a/b, and TIFY6 groups) were strongly induced by freezing either with or without cold acclimation, while they were not induced by the chilling treatment.

**Fig. 6:**
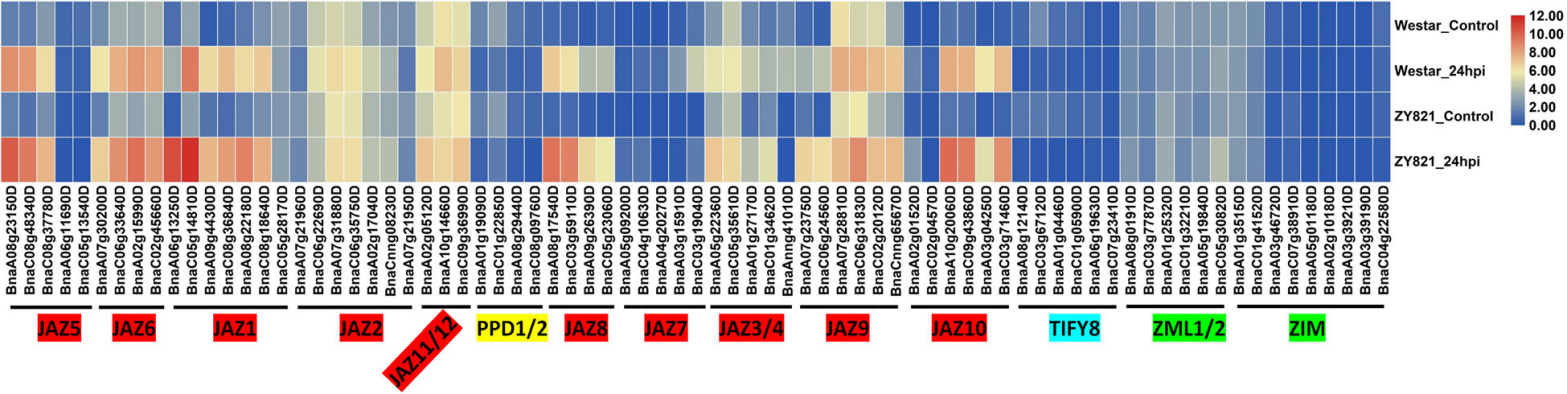
Expression of *BnaTIFY* genes in *B. napus* infected with *S. sclerotiorum*. The expression levels of each gene (log_2_ (FPKM values)) in each sample, as indicated by different color rectangles

*S. sclerotiorum*, a prototypical necrotrophic fungus, causes Sclerotinia stem rot, one of the most devastating diseases of *B. napus* [53]. Studies have shown that JA signaling and JAZ play important roles in the resistance of plants to necrotrophic and hemibiotrophic fungal pathogens [21, 22]. We analyzed the patterns of expression of *BnaTIFY* genes in *B. napus* leaves infected with *S. sclerotiorum* (Additional file 13, data from GEO: GSE81545) [54]. Almost all of the JAZ subfamily members were strongly induced by infection with *S. sclerotiorum* at 24 h post inoculation (hpi) in *B. napus* leaves, and the change-fold (24 hpi/control) in a tolerant cultivar of *B. napus* (*B. napus* cv. Zhongyou 821) was higher than that in a susceptible one (*B. napus* cv. Westar) (Fig. 6), while the PPD, TIFY and ZML subfamilies were slightly or not induced by infection with *S. sclerotiorum*.

**Fig. 7:**
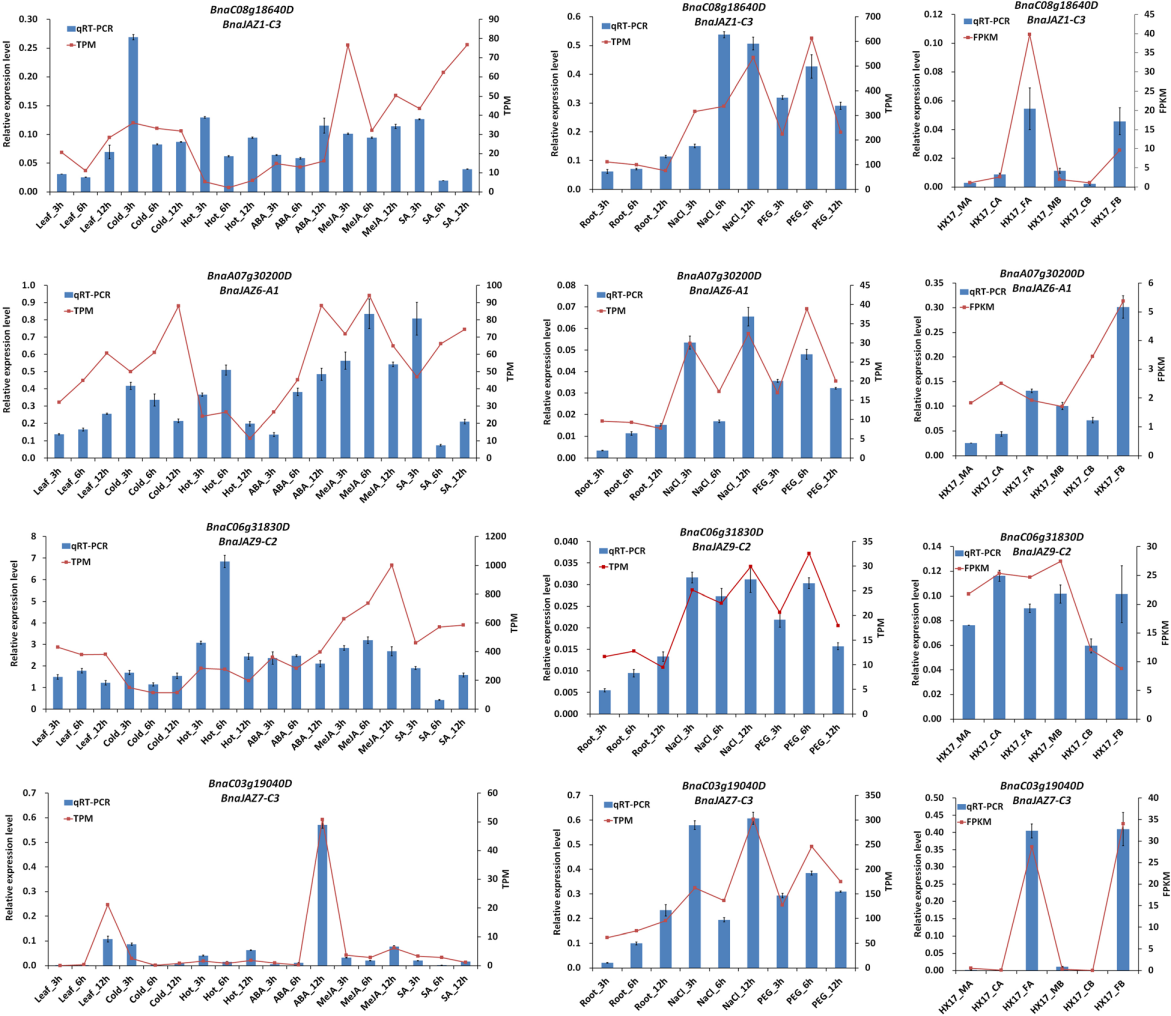
qRT-PCR analysis of expression patterns of *BnaTIFY* genes under different abiotic stresses and hormone treatments. Compare of qRT-PCR data (blue bar) and RNA-Seq data (red line). The relative qRT-PCR expression level is shown on the left y-axis. *BnaActin* was used as an endogenous reference gene. The relative transcript level was determined and normalized using the reference level and averaged over the three technical replicates. The FPKM or TPM from the RNA-Seq data is indicated on the right y-axis

We determined the abundance of transcripts of four *BnaTIFY* genes (*BnaJAZ1-C3*, *BnaJAZ6-A1*, *BnaJAZ9-C2*, and *BnaJAZ7-C3*) under abiotic stresses and phytohormone treatments to confirm the results of the RNA-Seq data using qRT-PCR. As shown in Fig. 7, all four genes were induced by NaCl and PEG stress in roots and by heat stress in leaves. *BnaJAZ1-C3*, *BnaJAZ6-A1* and *BnaJAZ7-C3* were induced by cold and SA at 3 h post treatments and were upregulated by MeJA at all three times. Additionally, *BnaJAZ7-C3* was induced by ABA. Although the fold-changes in their expression detected by RNA-Seq did not exactly match those detected by qRT-PCR, the detected expression patterns were mostly consistent for all the selected genes, confirming the reliability of the RNA-Seq results. In summary, all of the results identify these genes as candidates for further study on the potential roles of *BnaTIFY* in multiple stress responses of *B. napus*.

### *Cis*-element analysis of TIFY promotors

The putative root-specific *cis*-elements (4), phytohormone-responsive *cis*-elements (6), environmental stress-responsive *cis*-elements (8) and transcriptional factor binding sites (4) in the 2000 bp region upstream sequences of each of *TIFY* gene were analyzed using PLACE (https://www.dna.affrc.go.jp/PLACE/?action=newplace) and PlantCARE (http://bioinformatics.psb.ugent.be/webtools/plantcare/html/) (Additional file 14). As shown in Additional file 14, the number of root-specific *cis*-elements in the *TIFY* genes from *B. rapa*, *B. oleracea*, An- and Cn-subgenomes of *B. napus* did not differ significantly, although the levels of expression of *BoJAZ* and *BnaJAZ* genes were higher than those of *BrJAZ* genes in the root (Fig. 3). The lack of correlation between patterns of expression and conserved promoter/gene structures of those *TIFY* orthologous gene pairs indicated the presence of heritable epigenetic modulations during allopolyploidization in *B. napus*. The CGTCA/TGACG-motif (*cis*-acting regulatory element involved in MeJA-responsiveness) and ABRE (*cis*-acting element involved in ABA responsiveness) are the largest number of phytohormone-responsive *cis*-elements, and the light-responsive and ARE (*cis*-acting regulatory element essential for the anaerobic induction) are the largest number of environmental stress-responsive *cis*-elements in the TIFY promotors. MYCCONSENSUSAT (the MYC binding site) contains the largest number of transcriptional factor binding sites in TIFY promotors.

## Discussion

TIFY proteins have proven to be important regulators in abiotic and biotic stress responses in plants. Since the *Arabidopsis* JAZ subfamily was identified as the vital repressor in JA signaling, increasing numbers of other JAZ proteins have been characterized as important stress-response regulators in various plant species, including *B. rapa*, *B. oleracea*, rice, cotton, tomato, poplar, grape, soybean, wheat, maize, rubber tree, Moso bamboo and purple false brome [36–51, 55–57]. However, no *B. napus* TIFY was isolated and identified over the whole genome.

**Fig. 8:**
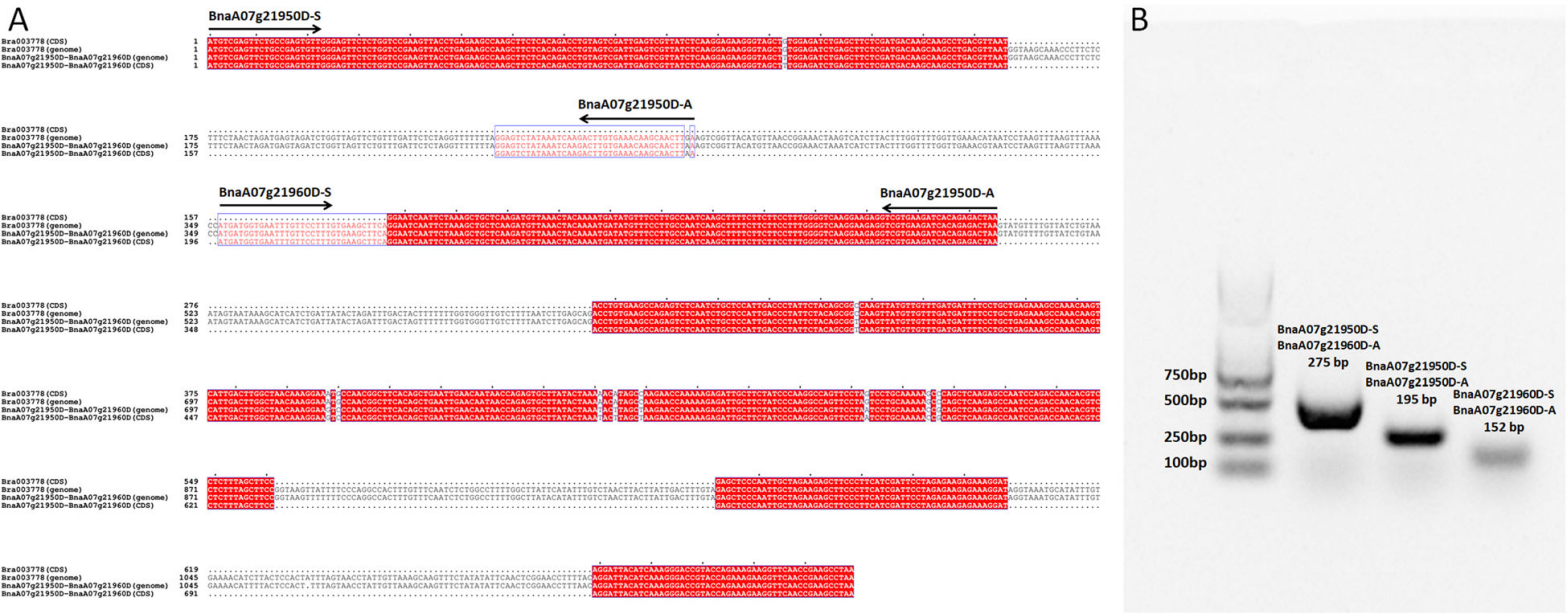
Sequence analysis of *Bra003778*, *BnaA07g21950D* and *BnaA07g21960D* (**a**) and PCR analysis (**b**). The blot of PCR production was cropped from the full gel image in Additional file 16 (**b**)

Previous studies indicated that a total of 36 and 36 TIFY proteins were identified in *B. rapa* accession Chiifu-401-42 [51] and *B. oleracea* var. *capitate* lines (02–12 and D134) [41]. In this study, we identified 77, 36 and 39 TIFY family genes in the genomes of *B. napus*, *B. rapa* and *B. oleracea* kale-like type TO1000DH, respectively (Additional file 1). In our study, three new TIFY genes were identified in *B. oleracea*, owing to the different versions of reference genomes. The results of a compared analysis between 36 *BoTIFY* genes in 02–12/D134 and 39 *BoTIFY* genes in TO1000 showed that the three new genes identified were *Bo1g119100* (*BoJAZ3/4–2*)*, Bo7g082470* (*BoTIFY8–3*) and *Bo9g152530* (*BoTIFY8–5*) in TO1000. Further analysis showed that the study by Liu [51] had failed to identify *BoJAZ* gene Bol030895 (C01:31,896,448..31,899,117) in 02–12 (the homologous gene locus in TO1000 was *Bo1g119100*). Four members from the JAZ subfamily from *B. napus* contained only the Jas domain, and one member contained only the NT domain (Additional file 1). We hypothesized that all of them were new genes that had recently mutated from typical TIFY members. For example, *BnaA07g21950D* (only contained in the NT domain) was located 81 bp upstream of *BnaA07g21960D* (a TIFY-Jas type *BnaJAZ*); the two members may have diverged from a NT-TIFY-Jas type *BnaJAZ*. To prove this hypothesis, we analyzed the genome sequences of *BnaA07g21950D-BnaA07g21960D* and the homologous chromosomal regions in *B. rapa*. As shown in Fig. 8a, the stop codon of *BnaA07g21950D* and start codon of *BnaA07g21960D* were located in the region homologous to the first intron of *Bra003778*. Therefore, we hypothesized that there may have been a longer transcript similar to *Bra003778* in *B. napus*. We designed two pairs of primers (BnaA07g21950D-S/A and BnaA07g21960D-S/A) for PCR using the cDNA of ZS11 (*B. napus* L. cv. Zhongshuang 11) leaves. As expected, the prospective PCR products were obtained by the two pairs of primers. Interestingly, there was also a PCR product (275 bp) identified using BnaA07g21950D-S and BnaA07g21960D-A (Fig. 8b and Additional file 16). Thus, there are splice variants in this region in *B. napus*.

In *B. napus*, the An- and Cn-subgenomes were collinear to the corresponding diploid Ar and Co genomes, respectively [35]. The majority of An-Ar and Cn-Co orthologous gene pairs have similar chromosomal locations. The chromosomal locations of most *TIFY* genes in the *B. napus* An- and Cn-subgenomes were similar to those of the orthologous *TIFY* genes in *B. rapa* and *B. oleracea*, respectively (Additional file 3). For example, with the exception of *Bra030986*, which has no orthologous *TIFY* genes in the *B. napus* An-subgenome, the distribution of all 34 *TIFY* genes in *B. rapa* were similar to those of the orthologous *TIFY* genes in *B. napus* An-subgenome (Additional file 3) [41]. Similar to the *B. rapa* vs *B. napus* An-subgenome, the distribution of *TIFY* genes in *B. oleracea* was similar to those of the orthologous *TIFY* genes in the *B. napus* Cn-subgenome (Additional file 3) [51].

Unlike chromosomal locations and gene structures, the patterns of expression of the *TIFY* genes varied in *B. napus*, *B. rapa* and *B. oleracea*. The JAZ subfamily genes were predominantly expressed in flower buds in *B. rapa* [41], while the majority of JAZ subfamily members were predominantly expressed in roots in *B. napus* (Fig. 3) and *B. oleracea* [51]. This difference indicated that the spatiotemporal patterns of expression of *BnaJAZ* genes were more similar to those of *BoJAZ* than to *BrJAZ*, regardless of whether the *BnaJAZ* members were from the *B. napus* An- or Cn-subgenomes. A common trait that is similar to the expression of *TIFY* genes in other species is that the JAZ subfamily genes in *B. napus* (Fig. 4), *B. rapa* [41] and *B. oleracea* [51] were highly induced by JA/MeJA. The *BrJAZ* genes were highly regulated by cold, salinity, drought and ABA, but not by SA [41]. Only a few of the *BoJAZ* genes were induced by treatment with SA and ethylene. Most of the *JAZ* genes in *B. napus* were upregulated by NaCl, PEG and SA but not by ABA and ethylene (Fig. 4). In contrast to *BrJAZ*, the *BnaJAZ* genes were strongly induced by freezing, while they were not induced by chilling (Fig. 5). The results indicated that there was differentiation in the pattern of expression of JAZ family genes in Brassicaceae species.

In this study, we found that the TIFY, PPD and ZIM/ZML subfamilies were low-expression genes and were not induced by treatment with phytohormones in *B. napus* (Figs. 3, 4 and 5). However, the *BnaJAZ* genes were predominantly expressed in *B. napus* roots and responded to multiple abiotic/biotic stresses and treatment with phytohormones. Two pairs of genes (*BnaJAZ7-A3*/*BnaJAZ7-C3* and *BnaJAZ1-A2*/*BnaJAZ1-C2*) and four pairs of genes (*BnaJAZ5-A1/BnaJAZ5-C1*, *BnaJAZ1-A1/BnaJAZ1-C1*, *BnaJAZ8-A1/BnaJAZ8-C1*, and *BnaJAZ10-A2/BnaJAZ10-C2*) were induced to extremely high levels by the freezing treatment (> 100-fold to > 300-fold compared with the control) and infection with *S. sclerotiorum* (by > 500-fold to > 5000-fold compared with the control), respectively. They are valuable candidate genes for the genetic improvement of tolerance of plants to freezing stress and *S. sclerotiorum* infection in *B. napus*. Notably, the freezing-induced *BnaJAZ7-A3*/*BnaJAZ7-C3* genes were the only Jas domain-type members. Although the Jas domain has been identified as the essential degron in degradation of JAZ proteins [6, 12, 17, 18, 23], no functional characterizations of only Jas domain proteins has been reported in other plant species. Thus, there may be new and important functions of the only Jas domain proteins in JA-mediated freezing tolerance in *B. napus*. Thus, the *BnaJAZ* genes induced by freezing and infection with *S. sclerotiorum* can be edited using the CRISPR/Cas9 genome editing system to generate *B. napus* lines that are strongly adaptable to environmental stress.

## Conclusions

In this study, a total of 77, 36 and 39 TIFY family genes with 11 groups were identified in the genomes of *B. napus*, *B. rapa* and *B. oleracea*, respectively. Molecular evolutionary analysis showed that the TIFY gene structures were conserved in Brassicaceae species. Gene expression profiling and qRT-PCR revealed that different groups of *BnaTIFY* members have distinct spatiotemporal patterns of expression in normal conditions or when subjected to low temperature, heat, salt, PEG, ABA, MeJA, ethylene, SA treatments and infection with *S. sclerotiorum*. The JAZ subfamily members were predominantly expressed in *B. napus* roots and induced by NaCl, PEG, MeJA, SA, freezing treatments and infection by *S. sclerotiorum*, suggesting that they have vital roles in hormone signaling to regulate the tolerance to abiotic and biotic stresses in *B. napus*. The extensive annotation and expression analysis of the *BnaTIFY* genes contributes to our understanding of the functions of these genes in multiple stress responses and phytohormone crosstalk.

## Methods

### Plant materials and treatments

ZS11 (*B. napus* L. cv. Zhongshuang 11), early-maturing semi-winter rapeseed varieties (HX17 and HX58) were provided by Oil Crops Research Institute of the Chinese Academy of Agricultural Sciences.

ZS11 seeds were allowed to germinate, and then the seedlings were transplanted to pots containing soil or vermiculite. The growth conditions, hormone treatments, and abiotic stress conditions were as described previously [58].

For chilling and freezing treatments with or without cold acclimation, the seedlings of HX17 and HX58 were used. They were treated as described previously [52].

### Identification of TIFY in *B. napus*, *B. rapa* and *B. oleracea*

Eighteen AtTIFY proteins were used as query sequences for the search of *B. rape*, *B. oleracea* and *B. napus* TIFY proteins by BLASTP (E-value <1e-5) in *Ensembl genomes* (http://ensemblgenomes.org/). The conserved domains were characterized using InterPro (http://www.ebi.ac.uk/interpro/). The molecular weight, isoelectric point, and subcellular localization of TIFY proteins were predicted in *Ensembl genomes* and ProtComp 9.0 (http://linux1.softberry.com/). TBtools v0.6696 [59] was employed to analyze the gene structures of *TIFY* genes. The conserved motifs were analyzed with MEME (http://meme.nbcr.net/meme/cgi-bin/meme.cgi). The upstream 2000 bp DNA sequences of TIFY were analyzed using PLACE (https://www.dna.affrc.go.jp/PLACE/?action=newplace) and PlantCARE (http://bioinformatics.psb.ugent.be/webtools/plantcare/html/).

### Phylogenetic analysis of the TIFY proteins

Multiple sequence alignment of 170 TIFY proteins from *Arabidopsis*, *B. napus*, *B. rapa*, and *B. oleracea* were performed using ClustalW and a phylogenetic tree was constructed using the neighbour-joining (NJ) phylogenetic method in MEGA X [60] with 1000 bootstrap replicates.

### Nonsynonymous and synonymous substitution rate ratio (*Ka/Ks*)

The nonsynonymous substitution rate (*Ka*) and the synonymous substitution rate (*Ks*) between paralogous gene pairs were calculated using DnaSP (DNA Sequence Polymorphism) v684 [61].

### Chromosomal localization of *TIFY* genes

The position of *TIFY* genes on the chromosomes of *B. rapa*, *B. oleracea*, and *B. napus* were obtained using TBtools v0.6696 [59].

### RNA isolation and sequencing and gene expression analysis

The collected samples were sent to the sequencing cooperations of Sangon Biotech (Shanghai) Co., Ltd. and Novogene Co., Ltd. for RNA isolation, examination, and sequencing [52, 58]. qRT-PCR analysis was performed as described previously [58]. The primers used in this study were listed in Additional file 15.

### Heat map analysis

Heatmaps of the expression profile values (RPKM: Reads Per kb Per Million reads; TPM: Transcripts Per Million) of the Bna*TIFY* genes were generated by TBtools v0.6696 [59].

## Supplementary information

**Additional file 1 **Characteristics of TIFY in *B. napus*, *B. rapa* and *B. oleracea*.

**Additional file 2 **Syntenic analysis of *B. napus*, *B. rapa* and *B. oleracea* genomes. (A) Genome-wide syntenic relationships among A and C subgenomes in *B. napus* relative to the *B. rapa* (A genome: A01-A10) and *B. olereace* (C genome: C1-C9). Genic synteny blocks are connected by gray lines. (B) Syntenic dotplot between *Brassica napus*, *Brassica rapa*, *Brassica olereace* using whole-genome alignments by CoGe SynMap (https://genomevolution.org/coge/SynMap.pl).

**Additional file 3 **Distribution of *TIFY* genes on the *B. napus*, *B. rape* and *B. oleracea* chromosomes. A01–10: *B. rape* chromosomes (A); C01–09: *B. oleracea* chromosomes (C); chrA01–10: *B. napus* An-subgenome chromosomes; chrC01–09: *B. napus* Cn-subgenome chromosomes (B); Random means genes were randomly distributed to a specific chromosome. chrAnn and chrCnn were unanchored scaffolds that could not be mapped to a specific chromosome from the A- and C-subgenomes, respectively.

**Additional file 4.** 10 motifs were detected in BnaTIFY proteins using MEME.

**Additional file 5 **Gene structures of *Bo1g108240*, *BnaC01g32210D*-*BnaC01g32200D*, *Bra023900-Bra023899*, *BnaA01g25320D-BnaA01g25310D*.

**Additional file 6 **Sequence analysis of *Bo1g108240*, *BnaC01g32210D*, *BnaC01g32200D*.

**Additional file 7.** Sequence analysis of TIFY domain in BnaTIFY proteins.

**Additional file 8.** Sequence analysis of Jas and CCT domain in BnaTIFY proteins.

**Additional file 9 **Estimated Ka/Ks ratios of TIFY genes in *Arabidopsis, B. napus, B. rapa,* and *B. oleracea*.

**Additional file 10 **RNA-seq data of expression levels of *BnaTIFY* family genes across different tissues and organs.

**Additional file 11 **RNA-seq data of expression levels (TPM: transcriptions per millon) of *BnaTIFY* family genes under different stresses and phytohormones.

**Additional file 12 **RNA-seq data of expression levels of *BnaTIFY* family genes under cold and freezing treatments.

**Additional file 13 **RNA-seq data of expression levels of *BnaTIFY* in *B. napus* infected with *S. sclerotiorum*.

**Additional file 14 ***Cis*-elements present in the promoters of *BnaTIFY* genes in *B. napus*.

**Additional file 15.** List of primers used in this study.

**Additional file 16 **The full gel image of the productions of *BnaA07g21950D-BnaA07g21960D*.

## Data Availability

Additional data can be found in supplementary files. The RPKM value of *BnaTIFY* in leaves treated with chilling/freezing with or without cold acclimation were referenced from GEO (GSE129220). The RPKM of *BnaTIFY*s genes in *B. napus* leaves infected with *S. sclerotiorum* referenced from GEO: GSE81545).

## Abbreviations

JAZ: JASMONATE ZIM-domain
MeJA: Methyl jasmonate
SA: Salicylic acid
ABA: Abscisic acid
ET: Ethylene
PEG 6000: Polyethylene glycol 6000
ZIM/ZML: Zinc-finger Inflorescence Meristem: ZIM and ZIM-like
PPD: PEAPOD
RPKM: Reads Per kb Per Million reads
TPM: Transcripts Per Million
NT: N-terminal domain
Jas: JA-associated domain
