# Supplementary figures and images for: Genome-wide identification and functional analysis of the TIFY gene family in the response to multiple stresses in Brassica napus L
Source: BMC Genomics. 2020 Oct 22;21:736. doi: 10.1186/s12864-020-07128-2 (PMC7583176; doi:10.1186/s12864-020-07128-2)

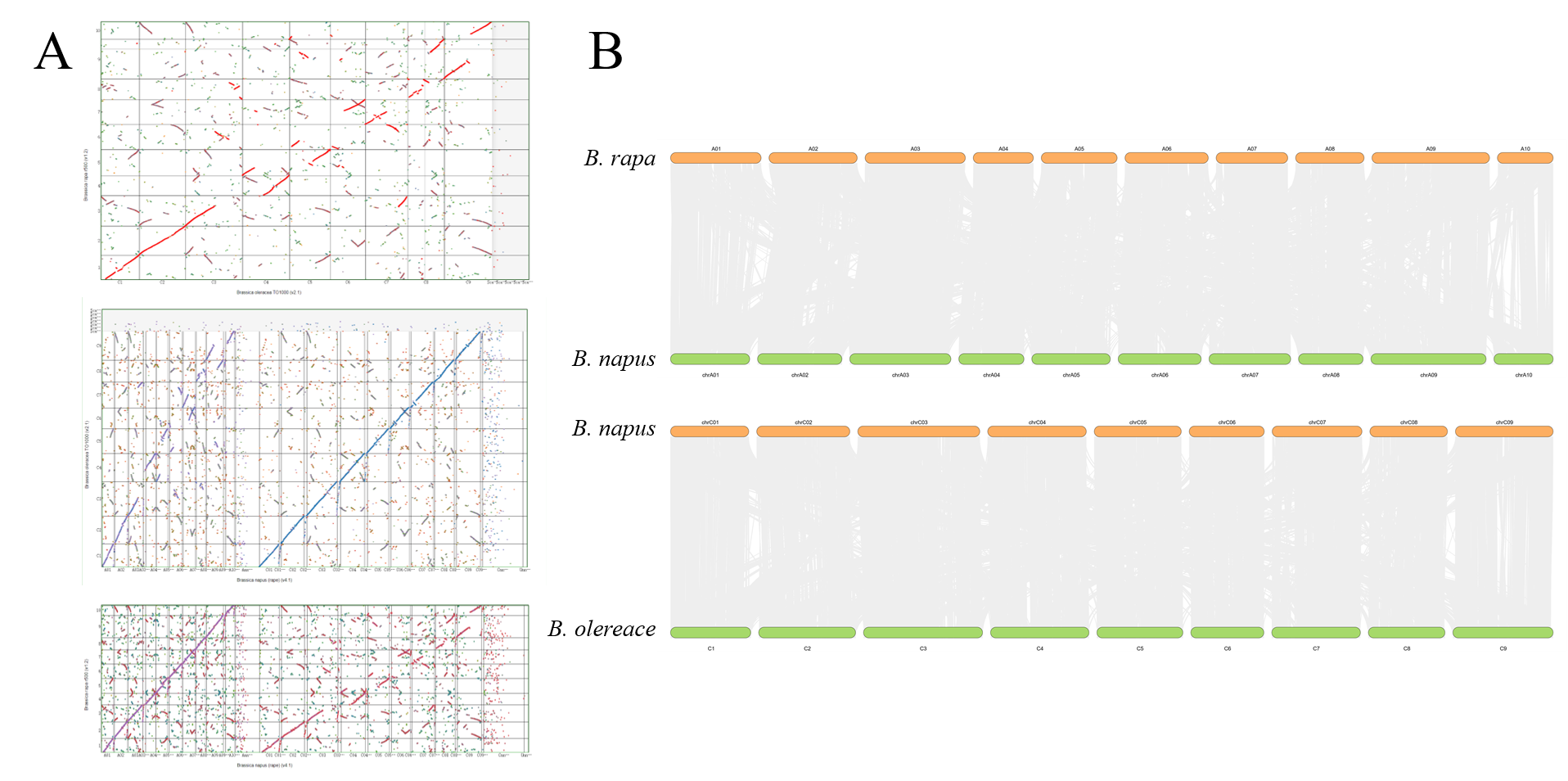

Supplement: Supplementary file 2 — Additional file 2 Syntenic analysis of B. napus, B. rapa and B. oleracea genomes. (A) Genome-wide syntenic relationships among A and C subgenomes in B. napus relative to the B. rapa (A genome: A01-A10) and B. olereace (C genome: C1-C9). Genic synteny blocks are connected by gray lines. (B) Syntenic dotplot between Brassica napus, Brassica rapa, Brassica olereace using whole-genome alignments by CoGe SynMap (https://genomevolution.org/coge/SynMap.pl). [file 12864_2020_7128_MOESM2_ESM.tif]

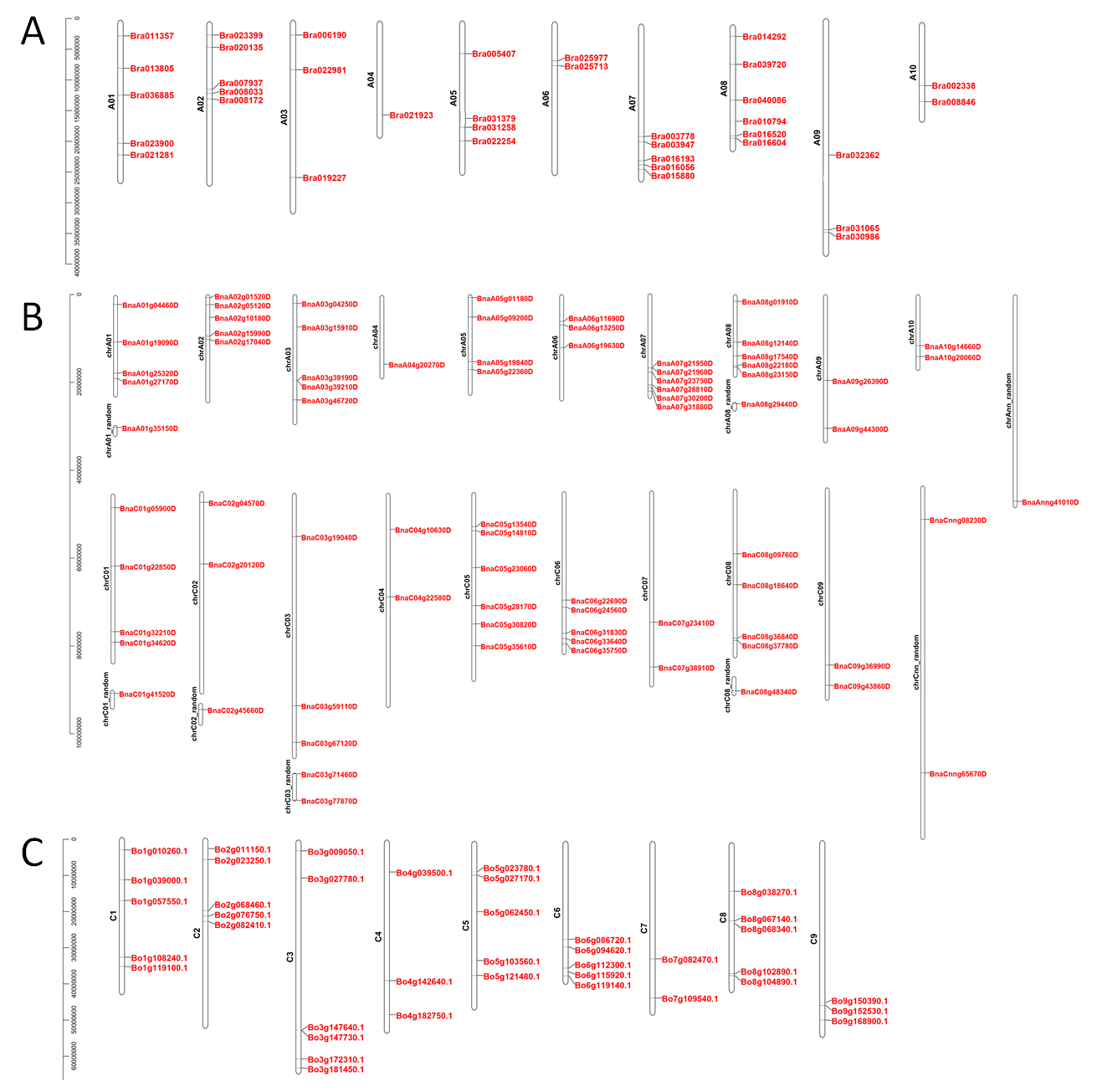

Supplement: Supplementary file 3 — Additional file 3 Distribution of TIFY genes on the B. napus, B. rape and B. oleracea chromosomes. A01–10: B. rape chromosomes (A); C01–09: B. oleracea chromosomes (C); chrA01–10: B. napus An-subgenome chromosomes; chrC01–09: B. napus Cn-subgenome chromosomes (B); Random means genes were randomly distributed to a specific chromosome. chrAnn and chrCnn were unanchored scaffolds that could not be mapped to a specific chromosome from the A- and C-subgenomes, respectively. [file 12864_2020_7128_MOESM3_ESM.jpg]

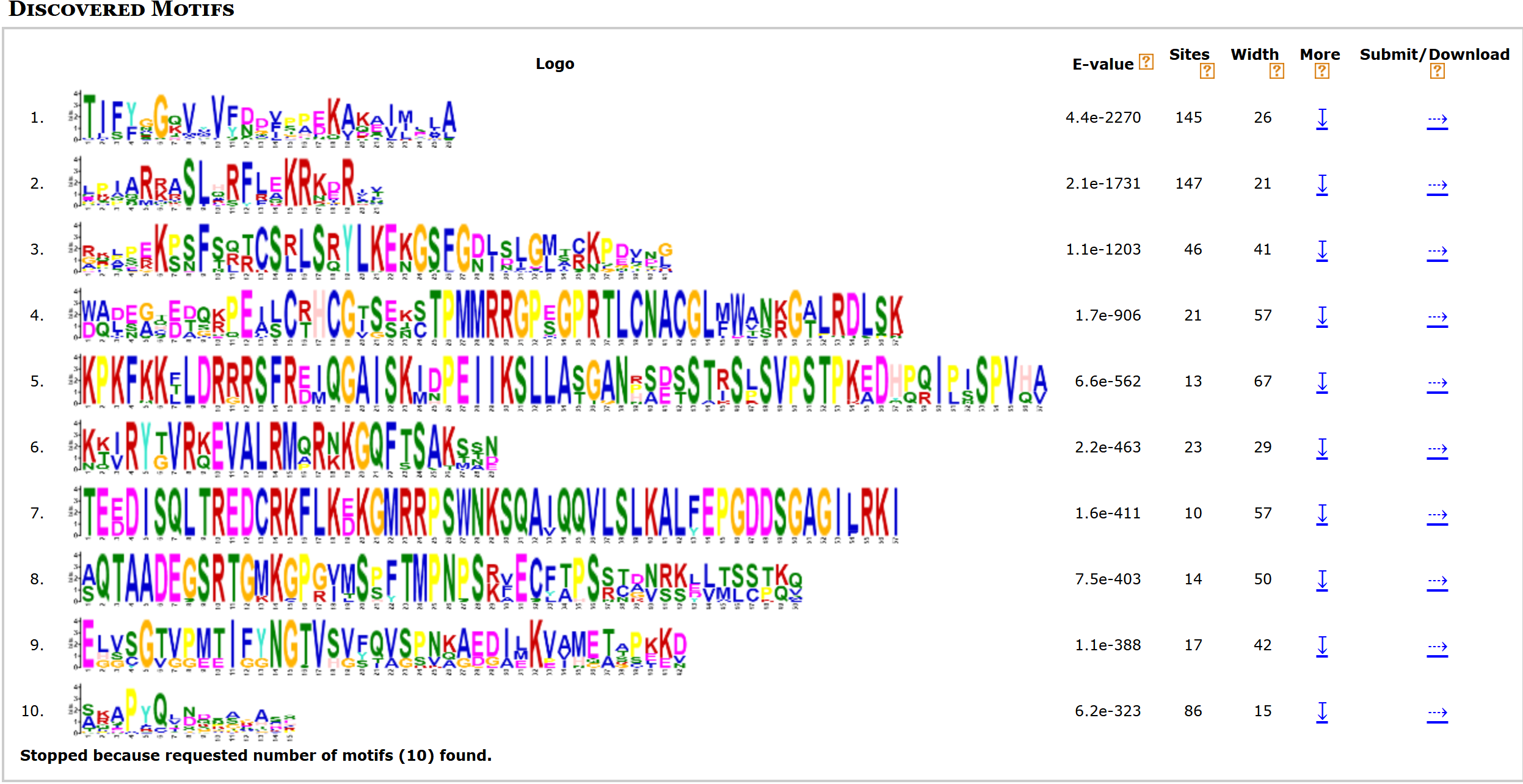

Supplement: Supplementary file 4 — Additional file 4. 10 motifs were detected in BnaTIFY proteins using MEME. [file 12864_2020_7128_MOESM4_ESM.png]

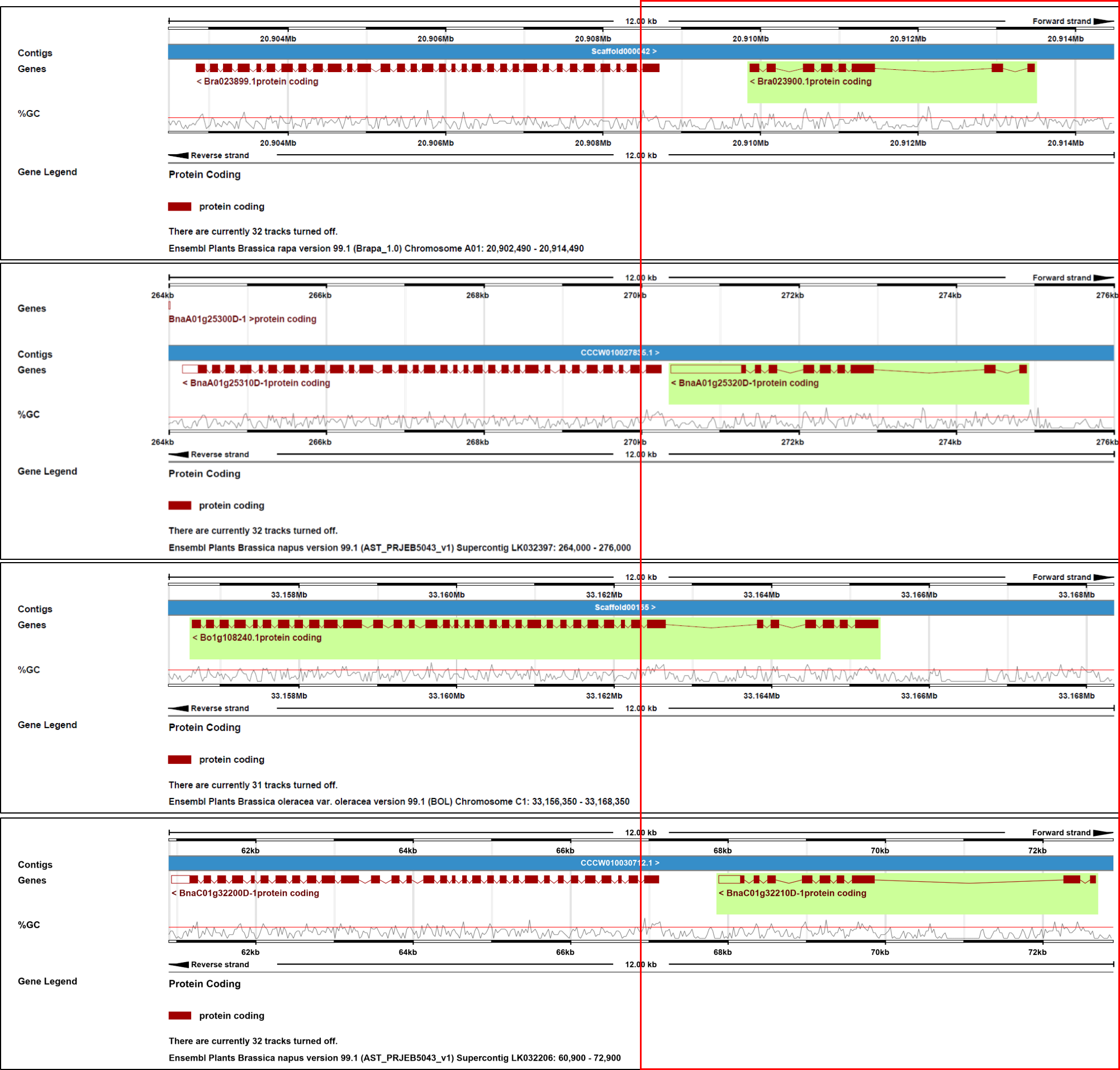

Supplement: Supplementary file 5 — Additional file 5 Gene structures of Bo1g108240, BnaC01g32210D-BnaC01g32200D, Bra023900-Bra023899, BnaA01g25320D-BnaA01g25310D. [file 12864_2020_7128_MOESM5_ESM.tif]

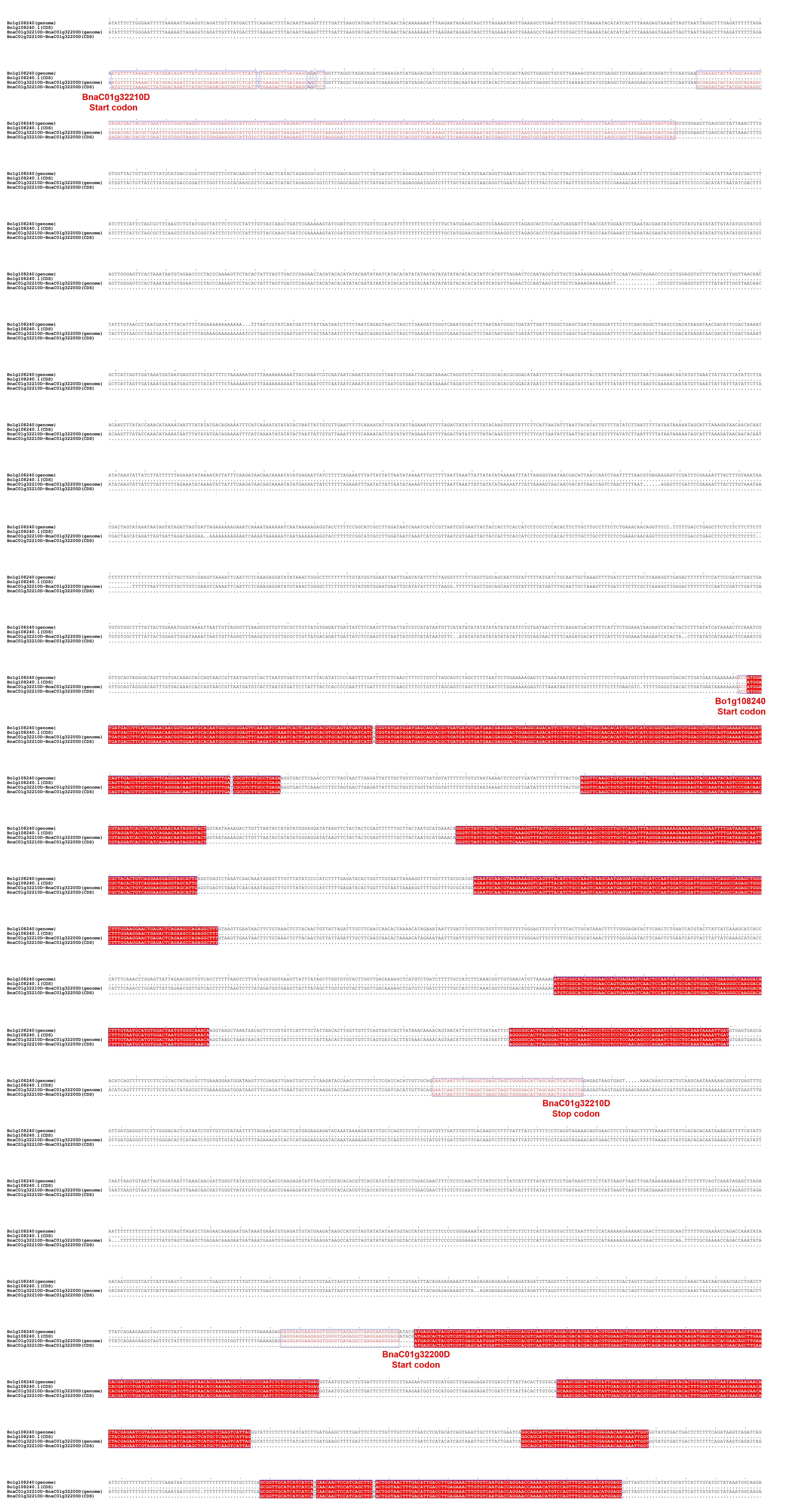

Supplement: Supplementary file 6 — Additional file 6 Sequence analysis of Bo1g108240, BnaC01g32210D, BnaC01g32200D. [file 12864_2020_7128_MOESM6_ESM.tif]

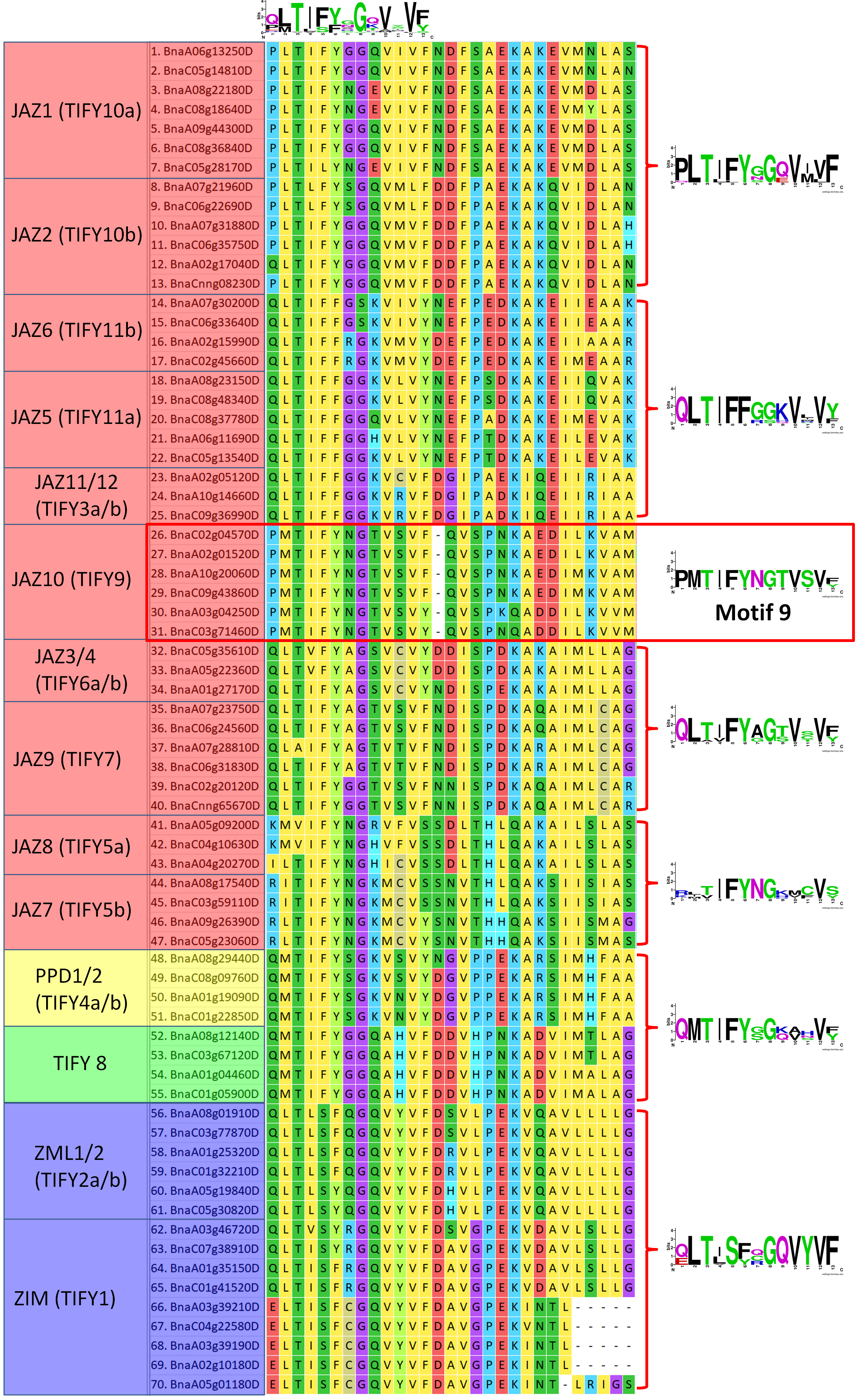

Supplement: Supplementary file 7 — Additional file 7. Sequence analysis of TIFY domain in BnaTIFY proteins. [file 12864_2020_7128_MOESM7_ESM.jpg]

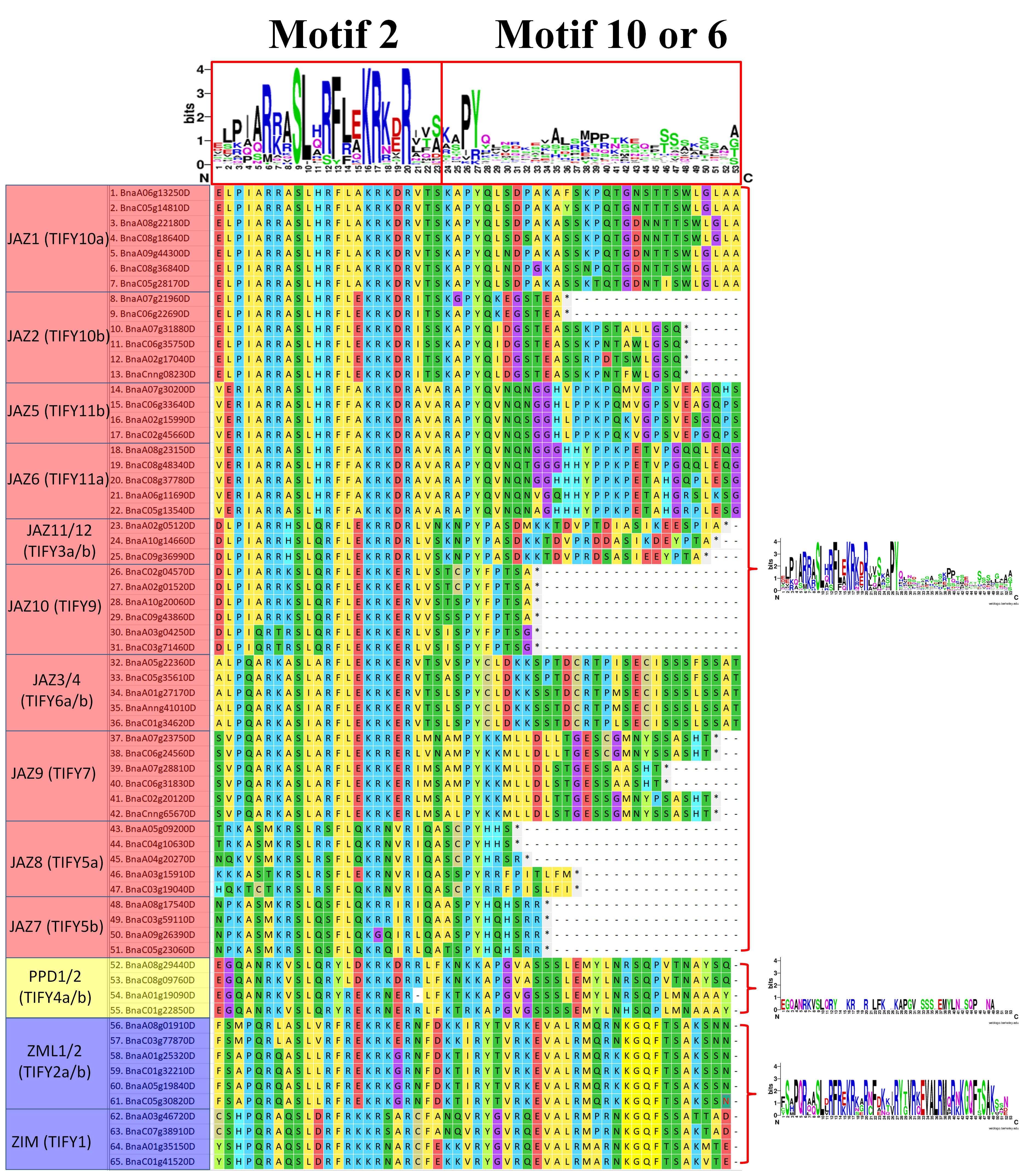

Supplement: Supplementary file 8 — Additional file 8. Sequence analysis of Jas and CCT domain in BnaTIFY proteins. [file 12864_2020_7128_MOESM8_ESM.jpg]

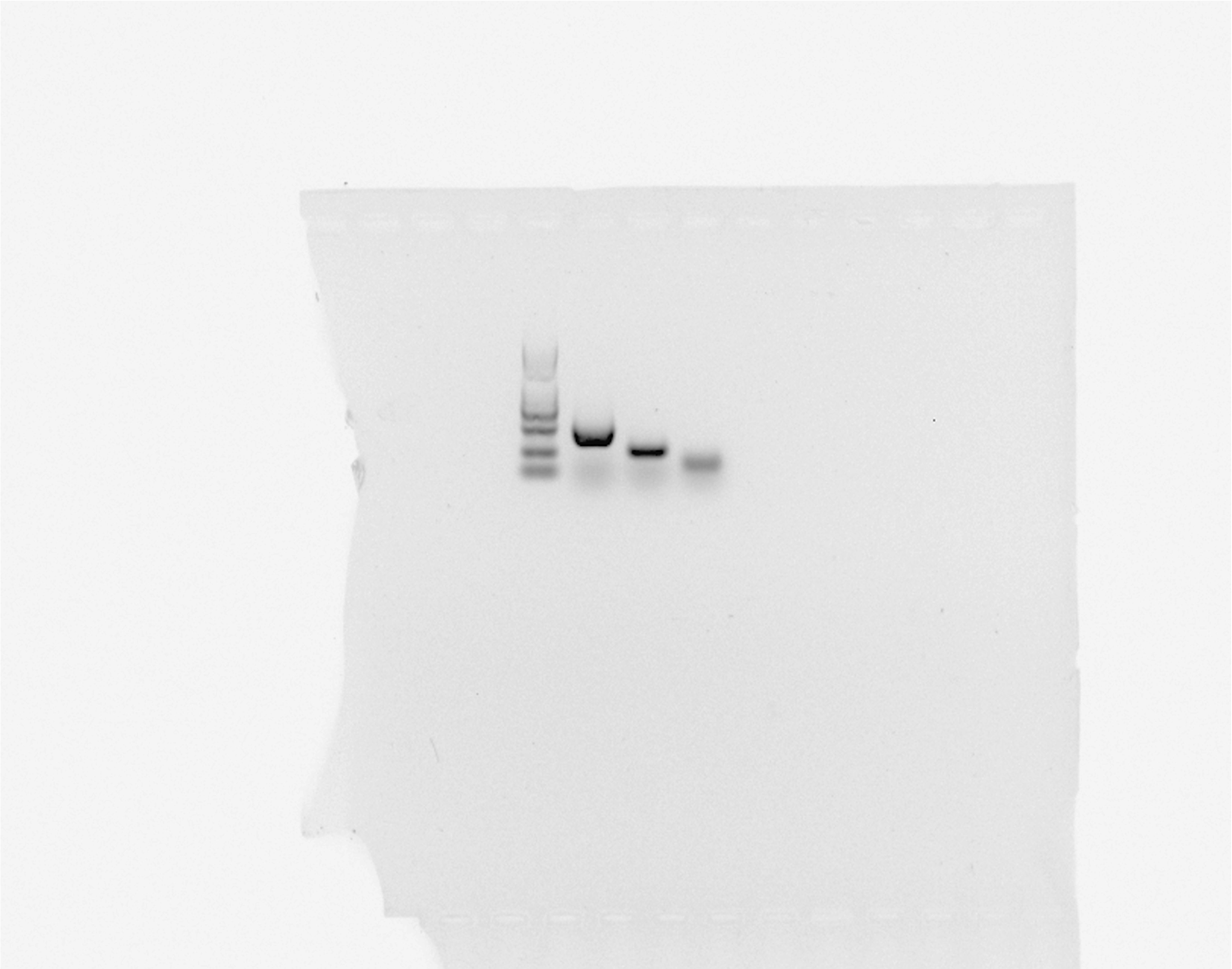

Supplement: Supplementary file 16 — Additional file 16 The full gel image of the productions of BnaA07g21950D-BnaA07g21960D. [file 12864_2020_7128_MOESM16_ESM.png]
